# The structure–function correlation analysed by OCT and full field ERG in typical and pericentral subtypes of retinitis pigmentosa

**DOI:** 10.1038/s41598-021-96570-7

**Published:** 2021-08-19

**Authors:** Ching-Wen Huang, Jung-Je Yang, Chang-Hao Yang, Chung-May Yang, Fung-Rong Hu, Tzyy-Chang Ho, Ta-Ching Chen

**Affiliations:** 1 Department of Ophthalmology, National Taiwan University Hospital, Taipei, Taiwan; 2 Department of Medical Education, National Taiwan University Hospital, Taipei, Taiwan; 3 Department of Ophthalmology, College of Medicine, National Taiwan University, Taipei, Taiwan; 4 Graduate Institute of Clinical Medicine, College of Medicine, National Taiwan University, Taipei, Taiwan

**Keywords:** Predictive markers, Medical research

## Abstract

To investigate the structure–function correlation analysed by full-field electroretinography (ffERG) and optical coherence tomography (OCT) in typical and pericentral subtypes of retinitis pigmentosa (RP). A retrospective, cross-sectional, observational study of right eyes was conducted. The primary analysis used ffERG data to compare the RP subtypes. The subgroup analysis was used to correlate the structure, analysed by OCT, and function, determined by ffERG. Linear regressions explored the relationship between best-corrected visual acuity (BCVA) and multiple parameters. A total of 188 eyes were included. Amplitudes of responses of rod, rod-cone, cone, and 30 Hz flicker of typical type were lower than those of pericentral and other types. In the subgroup analysis, 41 and 21 eyes of the typical and pericentral types were studied, respectively. The correlation between the estimated preserved photoreceptor area and all ffERG amplitude parameters were significant in the typical type, but not in pericentral type. Old age, decreased intact ellipsoid zone length, typical type, and thin central retinal thickness were negatively correlated with BCVA. Typical type RP developed more extensive degeneration and poorer BCVA compared to others. Strong structure–function correlation was found in typical type while not in pericentral type. OCT may be a useful tool for monitoring RP status in typical type, providing useful parameters for the prediction of BCVA.

## Introduction

Retinitis pigmentosa (RP) is an inherited disease characterised by the progressive loss of rods followed by cones^1^. The clinical presentation of RP includes night blindness, constriction of the visual field ("tunnel vision"), and vision loss. Several diagnostic tools, including visual field testing, optical coherence tomography (OCT), full-field electroretinogram (ffERG), multifocal electroretinogram (mfERG), microperimetry, and autofluorescence imaging, can be used to evaluate the progression of RP^2^. Since electroretinogram is an important tool to evaluate the function of rods and cones across the retina, it is an unreplaceable examination for RP, a type of rod-cone dystrophy. OCT is a useful tool for objectively monitoring RP progression within the posterior pole^3–6^. Additionally, OCT allows analysis of many parameters, including the residual ellipsoid zone (EZ) length^7–9^, preserved photoreceptor area^5^, and central retinal thickness (CRT)^10,11^, which are correlated to visual acuity (VA).

According to the morphological characteristics, RP could be mainly divided into two types: typical (T) and pericentral (P) type^12,13^. The T type has characteristic lesions originating from the far periphery and gradually extending to the central area of the retina. In contrast, the clinical characteristic of the P type is that the lesions mainly affect the surrounding area of the major temporal arcade on the retina, while the far periphery is relatively spared. As the disease progresses, the lesion may involve macular area^14^.

There has been relatively little research conducted on the structural and functional differences between different morphological patterns of RP. Hence, this study was conducted mainly for two purposes. One was to compare ffERG parameters, which represent photoreceptor function, in different subtypes of RP. The other was to correlate the functional and structural parameters in the different subtypes of RP.

## Materials and methods

### Study subjects

This retrospective and cross-sectional study adhered to the tenets of the Declaration of Helsinki and was approved by the National Taiwan University Hospital Research Ethics Committee. We retrospectively reviewed 188 patients with RP who visited the National Taiwan University Hospital between January 2012 and June 2020. All patients underwent comprehensive ocular examination, including best-corrected visual acuity (BCVA), slit-lamp biomicroscopy, funduscopic examination, fundus autofluorescence (FAF) imaging, visual field, and International Society for Clinical Electrophysiology of Vision (ISCEV) standard ffERG^15^. In addition, the patients included in the subgroup analysis underwent SD-OCT. All the above-mentioned examinations were conducted on the right eye to avoid inter-eye correlation. Information on age, sex, genetic diagnosis, major ocular disease, and operation history was also collected. The exclusion criteria were lack of data, macular edema due to RP, and concomitant ocular disease that could affect VA.

### Genetic diagnosis

After obtaining informed consent, the blood samples of patients were collected and genomic DNA from peripheral blood mononuclear cells were extracted. We performed genetic testing on patients’ genomic DNA via probe capture-based next-generation sequencing (NGS) approach targeting 212 IRD-related genes (Supplemntal Table 1) selected from the RetNet database (https://sph.uth.edu/retnet/), OMIM database (https://www.ncbi.nlm.nih.gov/omim), and publications (PubMed search queries: hereditary retinal dystrophy). (Supplemental Table 1) The detailed protocol was described in our previous study^16^.

**Table 1:** Demographic data of electroretinogram (ERG) analysis in different subtypes of RP.

Type	Typical (T)	Pericentral (P)	Other (O)	
Eyes	90	74	24	
Sex(F:M)	58:42	54:46	41:59	Chi square: 0.371
Age (years)	43.3^a^ (range: 15–78)	48.7^b^ (range: 26–74)	44.4^ab^ (range: 18–74)	ANOVA: *p* = 0.048* Sidak test: P&T *p* = 0.045*
Macular lesions (%)	36.7%^b^	63.5%^a^	66.7%^a^	Chi square + Bonferroni correction: D & P > T

*F* female, *M* male, *ANOVA* analysis of variance.

^a, b^Means within a row with different superscripts are different at *P* < 0.05.

*p < 0.05.

### Classification of RP subgroup

The diagnosis of RP was made by funduscopic examination and ffERG. The classification into morphological subtypes was based on visual field and FAF findings. A characteristic feature of the typical (T) type is that the lesions originate from the far periphery and extend to the central area of the retina. On the contrary, the clinical characteristic of the pericentral (P) type is that the lesions usually affect the surrounding area of the major temporal arcade on the retina (5–30°), while the far periphery is spared, corresponding to an annular scotoma^12,13,17^. If the FAF findings do not match the characteristics of T or P type, it will be classified in the other (O) type. There is no obvious border between the healthy retina and the lesion in the O type. This type may indicate early disease course before the development of the more common types seen in the T or P type. Each subject can be further categorized into with or without macular involvement.

### Acquisition of data

BCVA was measured using Logarithmic Landolt “C” Eye Chart and then converted to logarithm of the minimum angle of resolution (logMAR). Full-field ERG recordings were obtained according to the ISCEV standards with few modifications including applying narrow band-pass filter (for 30 Hz flicker) and computer averaging (n = 20 sweeps) to expend the detection limit using Utas Visual Electrodiagnostic system^18^. Full-field ERG amplitude parameters, including dark-adapted 0.01 ERG (rod-driven response), b wave of dark-adapted 3.0 ERG (rod and cone-driven response), b wave of light-adapted 3.0 ERG (cone-driven response), and 30 Hz flicker (cone-driven response) were collected. If the amplitudes were less than 0.05 μV for 30 Hz flicker and less than 1 μV for the other parameters, they would be considered nondetectable, and were set to the value of 0.05 μV and 1 μV, respectively^19^. SD-OCT was performed using an OCT machine (RTVue XR Avanti, Optovue, Inc.). CRT was recorded using an OCT thickness map. The lengths of the intact EZ in four directions (nasal, temporal, superior, and inferior) were determined by measuring the distance from the center point to the end of the EZ in each direction. The estimated preserved photoreceptor area was calculated using the ellipse area formula (π × horizontal EZ length × longitudinal EZ length).

### Statistical analysis

To reduce positive skewness while comparing ffERG among different types, ffERG amplitudes were converted to logarithmic form. For comparing the mean age and mean log ERG amplitude parameters differences between different subtypes of RP, a one-way analysis of variance and a Sidak test were used. Sex and macula involvement percentages were also compared between the three subtypes by a chi-square test followed by post hoc analysis with Bonferroni correction.

In the subgroup analysis of the T and P types without macular involvement, age, average length of intact EZ, estimated preserved photoreceptor area, and CRT were compared using a t-test and sex distribution with a chi-square test. Linear regression was applied to determine the relationship between the estimated preserved photoreceptor area and ffERG amplitude parameters. To analyse the variability among the EZ lengths in four directions, we calculated the intraclass correlation coefficient (ICC) between the intact EZ length in these directions in an individual and demonstrated its corresponding 95% confidence interval (CI).

The relationships between BCVA and age, sex, average length of intact EZ, CRT, and ffERG amplitude parameters were examined using univariate and multivariate linear regression.

A *P*-value < 0.05 was considered statistically significant. All statistical analyses were performed using SPSS (Version 26, SPSS, Inc.). All figures were generated using Prism (Version 8, GraphPad Software, https://www.graphpad.com/scientific-software/prism/) and Keynote (Version 11.1, Apple Inc., https://www.apple.com/keynote/).

## Results

### Demorgraphic data in different subtypes of RP

A total of 188 right eyes of 188 patients were included in the primary analysis. Among the 188 eyes, T type, P type, and O type RP was identified in 90 (47.9%), 74 (39.4%), and 24 eyes (12.7%), respectively (Table 1). There was no difference in patient sex between the three groups. The mean ages of T type, P type, and O type were 43.3 years (range 15–78 years), 48.7 years (range from 26 to 74 years), and 44.4 years (range from 18 to 74 years), respectively. There was a statistically significant difference between the mean age of the T and P types. The percentage of macular lesions was lowest in the T (36.7%) compared to P (63.5%) and O types (66.7%).

Among the recruited patients, 146 subjects (77.7%) received genetic testing with 59 cases as T type, 65 cases as P type, and 22 cases as O type. The diagnostic rate through panel-based NGS test were 56% in T type, 55% in P type, and 82% in O type, respectively. High genetic heterogenity was shown in all the three subgroups. (Supplemental Fig. 1) The most common disease-causing genes in T and P type were *EYS, USH2A, CEP290* and *PRPF31* while disease-causing genes in O type were even more diverse.

**Figure 1:**
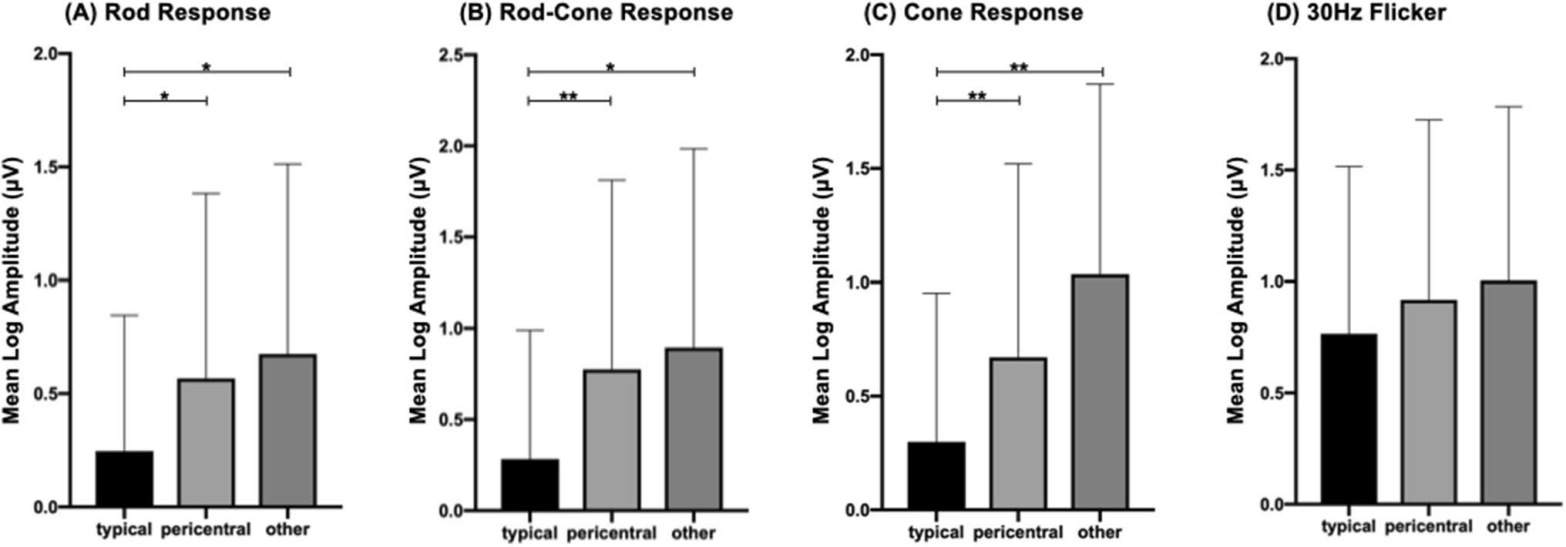
Different electroretinography response amplitudes of different subtypes of retinitis pigmentosa. (**A**) Mean log amplitudes of rod response were 0.246, 0.567, and 0.675 in typical, pericentral and other type, respectively. (**B**) Mean log amplitudes of rod-cone response were 0.283, 0.776, and 0.895 in typical, pericentral and other type, respectively. (**C**) Mean log amplitudes of cone response were 0.300, 0.671, and 1.035 in typical, pericentral and other type, respectively. (**D**) Mean log amplitudes of 30 Hz flicker were 0.766, 0.919, and 1.000 in typical, pericentral and other type, respectively. *p < 0.05; **p < 0.01 using analysis of variance followed by application of a Sidak test.

### Full field ERG characteristics in different subtypes of RP

The average log amplitudes of T type were lower than those of the P and O types in the dark-adapted 3.0 ERG (*p* = 0.004; T vs. O, *p* = 0.032; T vs. P, *p* = 0.016; P vs. O, *p* = 0.894), b wave of light-adapted 3.0 ERG (*p* < 0.001; T vs. O, *p* = 0.011; T vs. P, *p* = 0.002; P vs. D, *p* = 0.923), and dark-adapted 0.01 ERG (*p* < 0.001; T vs. O, *p* < 0.001; T vs. P, *p* = 0.006; P vs. O, *p* = 0.122). There was no statistically significant difference between the three subtypes in 30 Hz flicker (*p* = 0.280) (Fig. 1).

**Figure 2:**
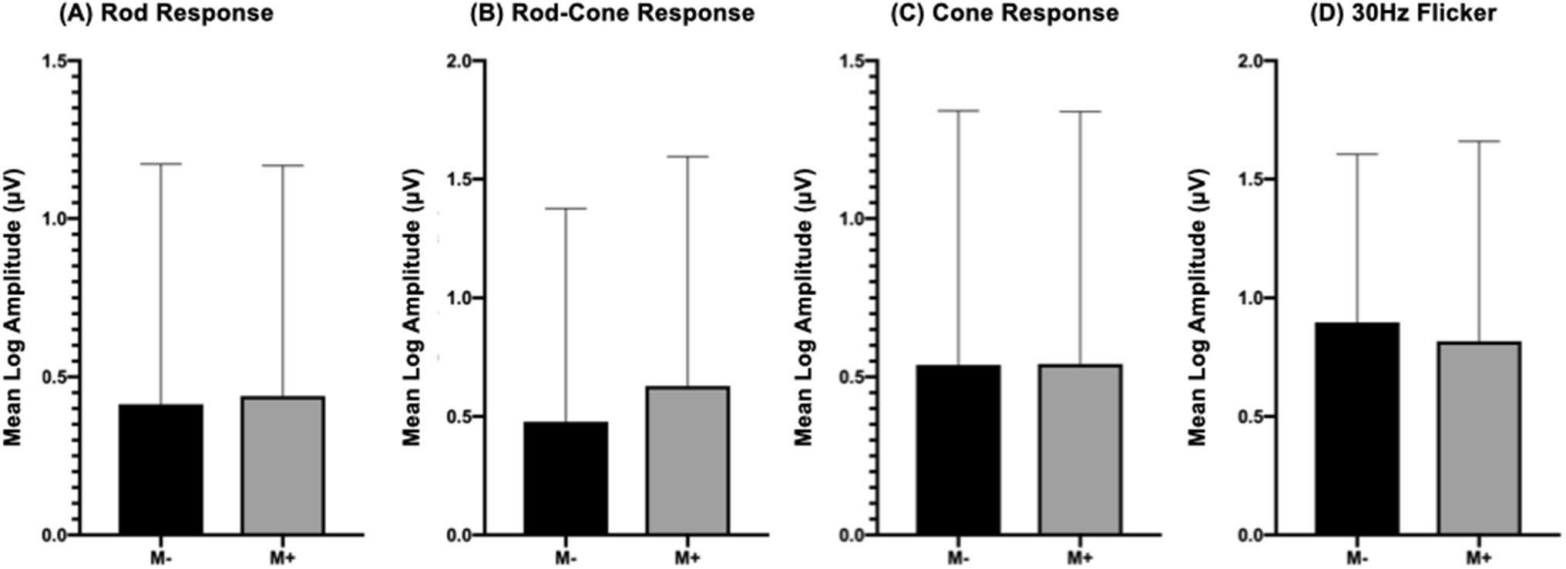
Different electroretinography response amplitudes of groups with or without macular involvement. (**A**) Mean log amplitudes of rod response were 0.413 and 0.440 in group without and with macular involvement, respectively. (**B**) Mean log amplitudes of rod-cone response were 0.478 and 0.629 in group without and with macular involvement, respectively. (**C**) Mean log amplitudes of cone responses were 0.538 and 0.541 in group without and with macular involvement, respectively. (**D**) Mean log amplitudes of 30 Hz flicker were 0.897 and 0.818 in group without and with macular involvement, respectively.*p < 0.05; **p < 0.01 using unpaired t-test.

On dividing all patients into two groups: those with (M + ; 92 eyes) or without (M-; 96 eyes) macular lesions, there were no statistical differences in sex (M + F:M = 49: 43; M–F:M = 53: 43; Chi-square: 0.789), age (mean age of M + 44.7 years; mean age of M- 46.5 years; *p* = 0.378), and all ffERG amplitude parameters between patients in M- and M + groups (Fig. 2). These results reflected that ffERG represent the summated condition of the retinal neurons that could be influenced by different morphological patterns of RP but not by macular lesions.

### Structure–function correlation in typical and pericentral types of RP

**Figure 3:**
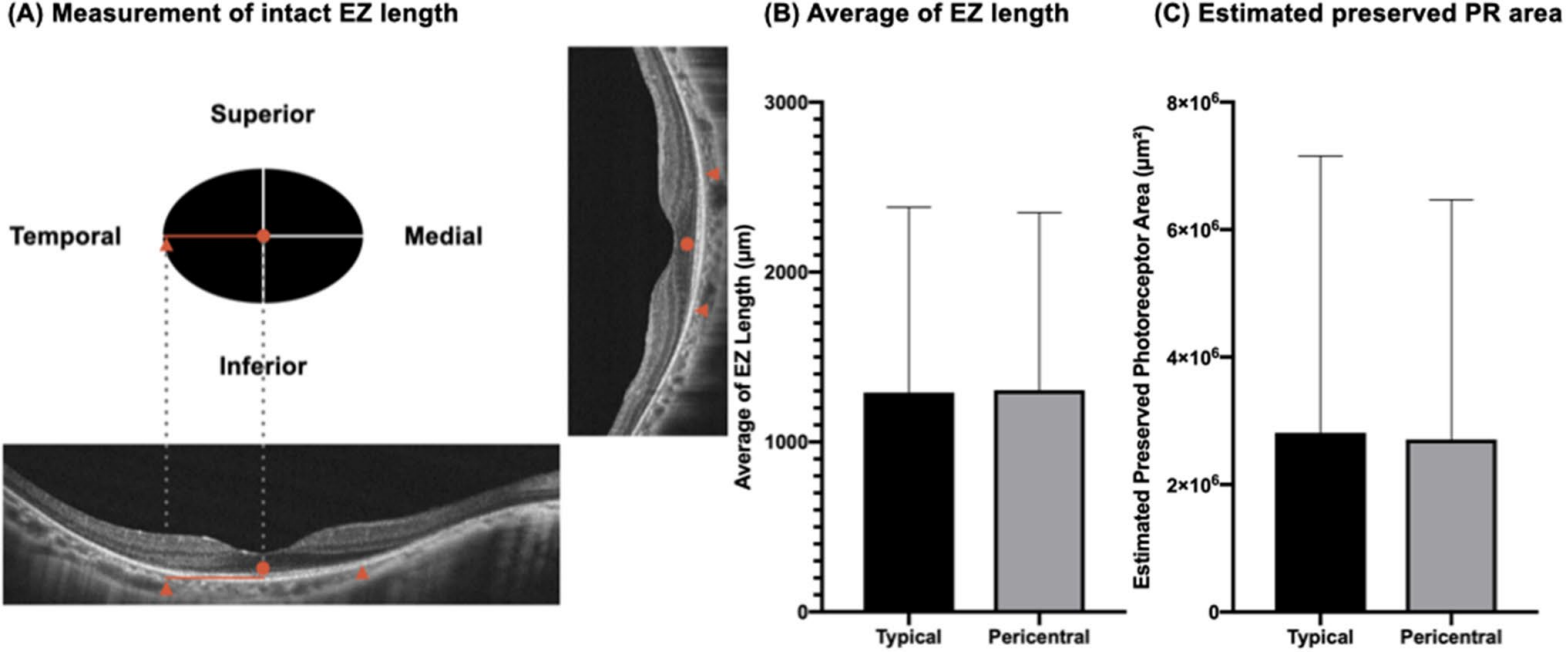
(**A**) Measurement of intact ellipsoid zone (EZ) length. The lengths of the intact EZ in four directions (nasal, temporal, superior, and inferior) were determined by measuring the distance from the center point to the end of the EZ in these directions. The estimated preserved photoreceptor area is calculated by the ellipse area formula (π x horizontal EZ length x longitudinal EZ length). (**B**) The average lengths of intact EZ were 1291.14 and 1305.18 µm in typical and pericentral types, respectively. (**C**) The estimated preserved photoreceptor areas were 2,814,352 µm^2^ and 2,707,140 µm^2^ in typical and pericentral types, respectively. *p < 0.05; **p < 0.01 using unpaired t-test.

As imaging techniques have advanced rapidly in recent years, we would like to explore the structure–function correlation in patients with RP. It was hard to measure the “intact EZ length” in the patients with macular lesion, which might present as fragmented or extensive disruption of EZ line on OCT. For minimising the possible errors when performing measurement, we chose only patients of T type without macular lesions (T–M type) and P type without macular lesions (P–M type) as study subjects. A total of 41 right eyes of 41 patients and 21 right eyes of 21 patients were included in the T–M and P–M types for the subgroup analysis, respectively. There were no statistical differences in sex (T–M F:M = 22:19; P–M F:M = 11:10; Chi-square: 0.931), age (mean age of T–M 46.4 years; mean age of P–M 48.6 years; *p* = 0.574) between T–M and P–M types. All recruited cases in this analysis had complete ffERG, BCVA, and OCT data. There were no statistically significant differences in the average of intact EZ length (T–M 1291.14 μm; P–M 1305.18 μm, *p* = 0.961), estimated preserved photoreceptor area (T–M 2,814,352 μm^2^; P–M 2,707,140 μm^2^, *p* = 0.924), and CRT (T–M 238.51 μm; P–M 232.10 μm, *p* = 0.743) between the two groups. There were also no statistically significant differences by mean log ffERG amplitudes (dark-adapted 0.01 ERG: T–M 0.280 vs. P–M 0.452, *p* = 0.373; b wave of dark-adapted 3.0 ERG: T–M 0.330 vs. P–M 0.507, *p* = 0.425; b wave of light-adapted 3.0 ERG: T–M 0.375 vs. P–M 0.579, *p* = 0.324; 30 Hz flicker: T–M 0.799 vs. P–M 0.880, *p* = 0.698) (Fig. 3).

**Figure 4:**
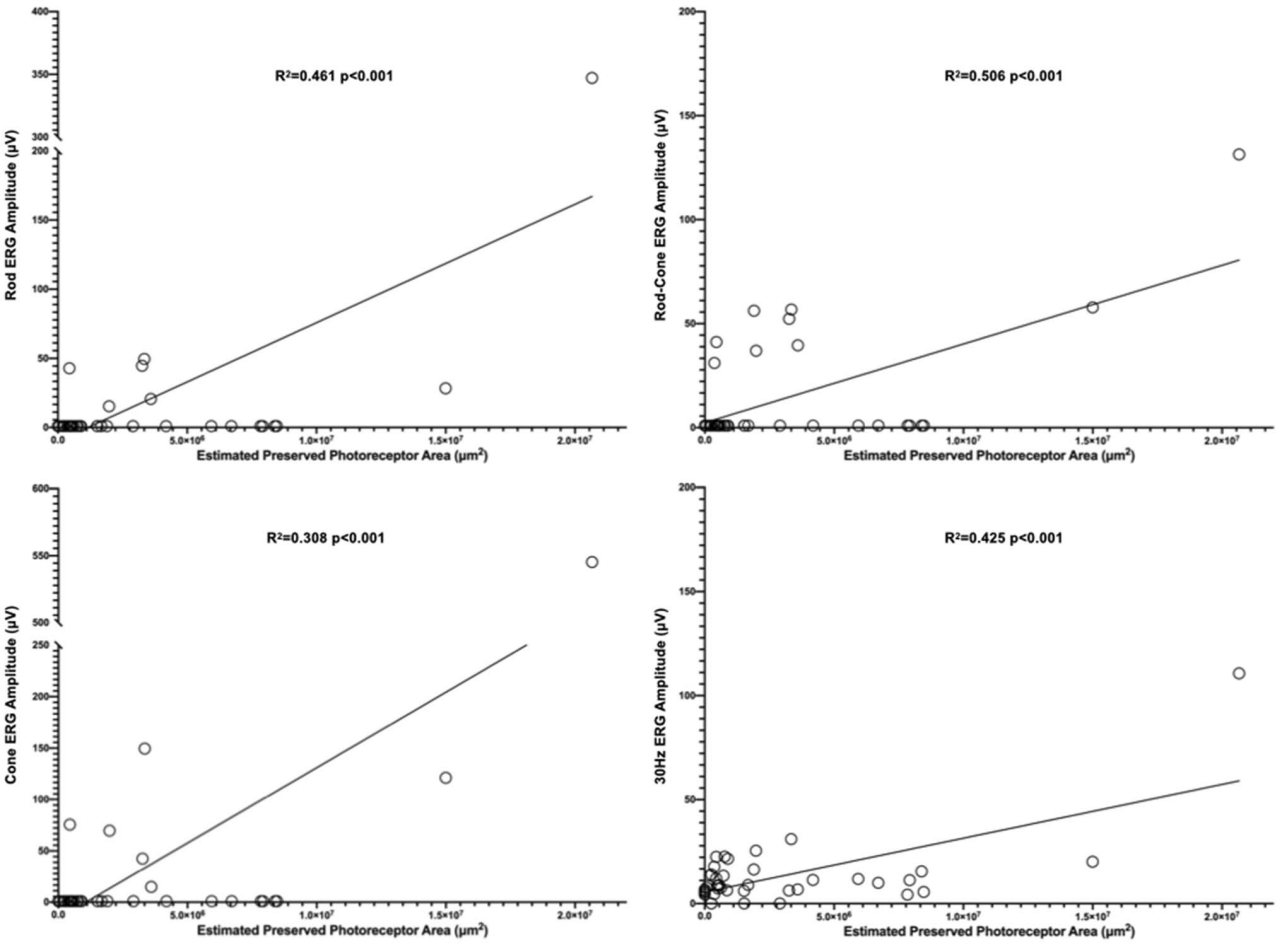
Scattered plot of all the ERG amplitude parameters and estimated preserved photoreceptor area for typical type.

We found a statistical correlation between all the ffERG amplitude parameters and the estimated preserved photoreceptor area in T–M type. (Fig. 4) This means that quantitative structural analysis could represent functional status. However, the correlations existed only in the T–M type and not in the P–M type (Table 2). To explore this difference, we conducted an interclass analysis, ICC, to evaluate the variations in EZ length from central fovea in four directions. The results of ICC for the T–M and P–M types were 0.958 (95% CI 0.933–0.975) and 0.856 (95% CI 0.745–0.931), respectively. This result demonstrated that the variability of the EZ length from the fovea to four directions in the P–M type was greater than that in the T–M type.

**Table 2:** Linear regression between electroretinography (ERG) parameters amplitude and estimated preserved PR area.

Electroretinography (ERG) parameters amplitude (μV) versus estimated preserved PR area (μm^2^)	Typical		Pericentral	
	*r*^2^	*P* value	*r*^2^	*P* value
Rod response	0.461	< 0.001*	0.000	0.906
Rod-cone response	0.506	< 0.001*	0.000	0.991
Cone response	0.380	< 0.001*	0.027	0.478
30 Hz flicker	0.425	< 0.001*	0.000	0.981

*p < 0.05.

We further explored the structure–function correlation of VA. The univariate linear regression analysis showed that the average EZ length (β = − 0.00027; *p* < 0.001) and CRT (β = − 0.006; *p* < 0.001) were correlated with BCVA. From the multivariate linear regression analysis using backward selection, age (β = 0.009; *p* = 0.011), average of intact ellipsoid zone (EZ) length (β = − 0.00016; *p* = 0.003), subtype (typical type, β = 0.254; *p* = 0.015), CRT (β = − 0.005; *p* < 0.001) were selected as the best explanatory variables for BCVA (Table 3).

**Table 3:** Univariate and multivariate analyses between logarithm of the minimum angle of resolution (logMAR) visual acquity (VA) and parameters.

Parameters	Univariate analysis				Multivariate analysis (Backward Selection)			
	Unstandardised		Standardised		Unstandardised		Standardised	
	β	S.E	Beta	*P* value	β	S.E	Beta	*P* value
Age	0.006	0.005	0.170	0.195	0.009	0.003	0.250	0.011*
Sex					N.S	N.S	N.S	N.S
Male	Ref							
Female	0.142	0.133	0.139	0.290				
Type								
Pericentral	Ref				Ref			
Typical	0.196	0.138	0.183	0.162	0.254	0.101	0.236	0.015*
AVG of EZ length	− 0.00027	0.00005	− 0.570	< 0.001*	− 0.00016	0.00005	− 0.338	0.003*
CRT	− 0.006	0.001	− 0.555	< 0.001*	− 0.005	0.001	− 0.435	< 0.001*
ERG rod-cone amplitude	− 0.001	0.001	− 0.179	0.170	N.S	N.S	N.S	N.S

*p < 0.05. *AVG of EZ length*average length of intact ellipsoid zone, *PR* photoreceptor, *CRT* central retinal thickness, *ERG* electroretinogram, *Ref*. reference, *N.S* not select.

## Discussion

RP is a heterogeneous disease in terms of both genotypes and phenotypes. In this study, we found that there are differences in ffERG findings between different subtypes of RP, reflecting the different extents of degeneration of photoreceptors. The P type, which was characterised as lesions mainly affecting the surrounding area of the major temporal arcade on the retina with the far periphery relatively spared, is considered a relatively rare type of RP and has great heterogeneity in the genetic phenotype^12,13,17,20^. Karali et al.^13^ found that the 30 Hz flicker of the P type was more preserved than that of the typical RP patients in previous studies^21,22^. In addition, Sandberg et al^12^. found that the annual progression in ffERG of the P type was lower than that of the T type as revealed by other studies^23^. In our study, we found a similar trend on comparing typical and pericentral types. Although the mean age of the T type group was slightly lower, their ERG amplitudes were significantly lower (except for 30 Hz flicker), suggesting that typical RP cause more extensive degeneration than pericentral. This result could also be explained by the fact that the T type had a larger area of involvement compared to the P type. (Supplementary Fig. 2).

In recent years, imaging techniques have progressed greatly. Non-invasive imaging, such as spectral-domain OCT in the posterior pole, provides quick and detailed anatomical evaluation. We were inquisitive about whether a structure–function correlation existed in the RP subtypes since it would be easier to evaluate the retinal function within seconds. Through analysis, we observed that the estimated preserved photoreceptor area using the ellipse equation (the product of horizontal preserved EZ length and vertical preserved EZ length, both measured by SD-OCT) was significantly correlated to whole retinal function analysed by ffERG in the T type. To the best of our knowledge, there is only limited research discussing the correlation between ffERG and OCT in patients with RP. Moschos et al. demonstrated that the average retinal thickness of the central ring was correlated to mfERG with borderline significance^24^. Abed et al. showed that there was a significant correlation between focal ffERG and EZ length in Stargardt disease^25^. However, the correlation between ffERG and OCT was not found in the P type. This might be because of two reasons: (1) the P type had inconsistent retinal involvement compared to the T type, where the preserved PR area was usually presented as a circle or elliptical shape. This assumption can be further proved by evaluating the consistency between the preserved EZ length in four directions using ICC analysis. (2) The P type was characterised by peripheral sparing in contrast to the T type. The estimated preserved photoreceptor area only represented the macular area; hence, it could not display the whole retinal function in the pericentral type. (Supplementary Fig. 1).

Many studies have discussed the relationship between VA and other parameters in patients with RP. The most important parameters were CRT^10,11^ and preserved EZ length^7–9^, which were significantly correlated with VA. Fahle et al.^26^ demonstrated that ffERG parameters were not correlated with VA. In our multivariate analysis, we found similar results as that of other studies. Moreover, we observed that subtypes were correlated to VA, and the T type was associated with poorer VA than that associated with the P type. The cone cell degeneration hypothesis could be a possible explanation for the above-mentioned findings. The pathogenesis of cone cell death in patients with RP remains unclear because the mutation in the rod is expressed in all such patients. Many findings described in previous studies, such as the dependence on trophic factors produced by rods, nutrient support by rods, oxidative stress, and pro-inflammatory microglial activation, have demonstrated that the pathogenesis of RP involves rod cell death followed by loss of cone cells^27^. We hypothesised that the T type have more diffuse rod cell involvement, which leads to more severe cone cell impairment (poor VA).

This is the first study to use function-structure relationships in analysing different morphological RP subtypes. Additionally, we elucidated that SD-OCT may be a useful tool for monitoring RP status in T type and provides good parameters, such as intact EZ length and CRT, for prediction of BCVA in patients with RP. However, there were still some limitations in this study, including its retrospective nature, non-consecutive tracking record, and use of partial data, such as the visual field test. Although this had a relatively large cohort, more cases were needed for better subgroup analysis. Further studies with larger sample sizes and complete data are warranted.

## Supplementary Information

Supplementary Figure 1 Legend.

Supplementary Figure 1.

Supplementary Figure 2 Legend.

Supplementary Figure 2.

Supplementary Table 1.

## Data Availability

The datasets generated during and/or analysed during the current study are not publicly available due to privacy and ethical concerns but are available from the corresponding author on reasonable request.
