# Supplementary material for: The structure–function correlation analysed by OCT and full field ERG in typical and pericentral subtypes of retinitis pigmentosa
Source: Sci Rep. 2021 Aug 19;11:16883. doi: 10.1038/s41598-021-96570-7 (PMC8376926; doi:10.1038/s41598-021-96570-7)
Supplement: Supplementary file 1 — Supplementary Figure 1 Legend. [file 41598_2021_96570_MOESM1_ESM.pdf]

The structure-function correlation analysed by OCT and full field ERG in typical and pericentral subtypes of retinitis pigmentosa.

Ching-Wen Huang[1], Jung-Je Yang[2], Chang-Hao Yang[1,3], Chung-May Yang[1,3], Fung-Rong Hu[1,3], Tzyy-Chang Ho[1,3], Ta-Ching Chen[1,4*]

[1]Department of Ophthalmology, National Taiwan University Hospital, Taipei, Taiwan
[2]Department of Medical Education, National Taiwan University Hospital, Taipei, Taiwan
[3]Department of Ophthalmology, College of Medicine, National Taiwan University, Taipei, Taiwan
[4]Graduate Institute of Clinical Medicine, College of Medicine, National Taiwan University, Taipei, Taiwan

* Corresponding author:
Ta-Ching Chen, MD
12F, No.7, Zhongshan S. Rd., Zhongzheng Dist., Taipei City 10002, Taiwan.
Tel: +886-2-23123456; ext: 63783
Email: tachingchen1@ntu.edu.tw

Supplemental Figure 1. tiff

Proportion of cases with indentified mutated genes in the typical, pericentral and the other type. Among the T type, 26(44%) cases was not identified with the pathogenic gene, which was same for 29 (44%) cases out of the P type and 4(18%) cases out of the other type. The pathogenic gene detected among the groups of the T and P type mostly were either EYS, USH2A, CEP290 or PRPF31. In contrast, the pathogenic gene detected in the group of the other types were varied.
