# Supplementary figures and images for: The structure–function correlation analysed by OCT and full field ERG in typical and pericentral subtypes of retinitis pigmentosa
Source: Sci Rep. 2021 Aug 19;11:16883. doi: 10.1038/s41598-021-96570-7 (PMC8376926; doi:10.1038/s41598-021-96570-7)

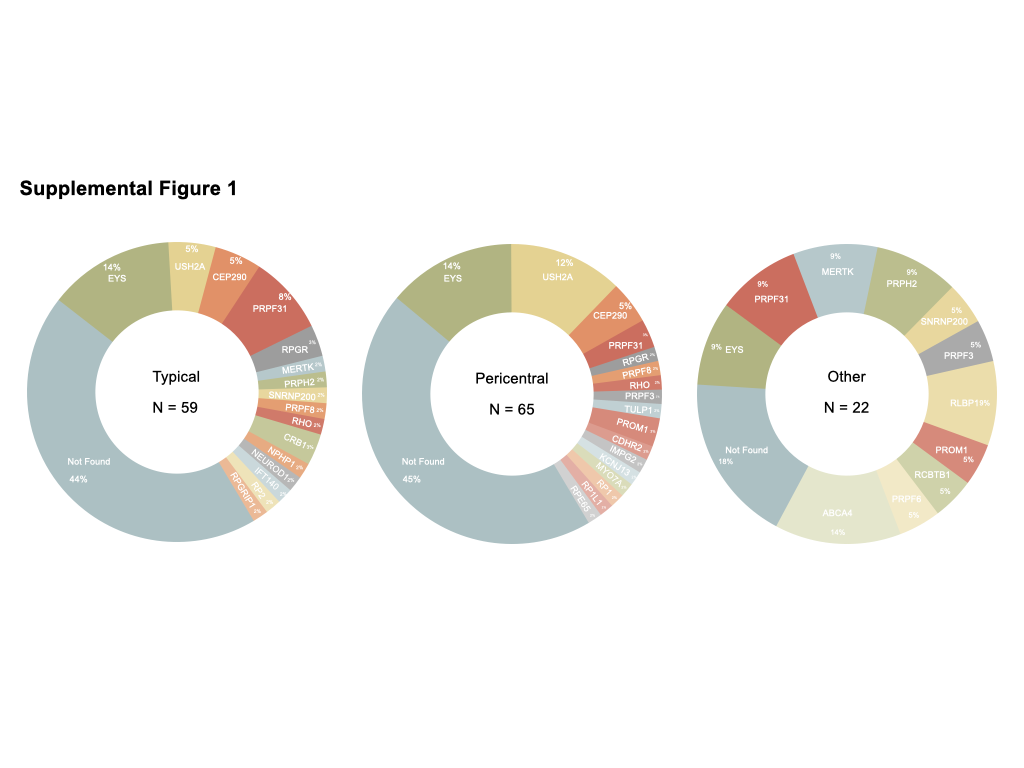

Supplement: Supplementary file 2 — Supplementary Figure 1. [file 41598_2021_96570_MOESM2_ESM.tiff]

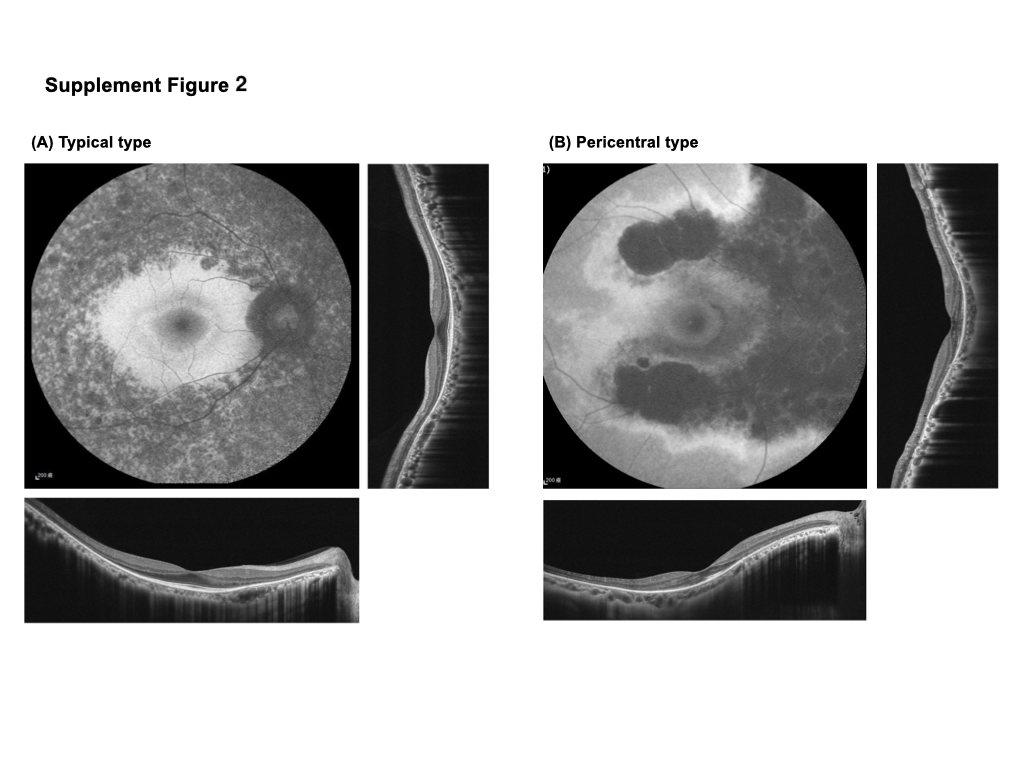

Supplement: Supplementary file 4 — Supplementary Figure 2. [file 41598_2021_96570_MOESM4_ESM.tiff]
