# Supplementary material for: The structure–function correlation analysed by OCT and full field ERG in typical and pericentral subtypes of retinitis pigmentosa
Source: Sci Rep. 2021 Aug 19;11:16883. doi: 10.1038/s41598-021-96570-7 (PMC8376926; doi:10.1038/s41598-021-96570-7)
Supplement: Supplementary file 3 — Supplementary Figure 2 Legend. [file 41598_2021_96570_MOESM3_ESM.pdf]

The structure-function correlation analysed by OCT and full field ERG in typical and pericentral subtypes of retinitis pigmentosa.

Ching-Wen Huang[1], Jung-Je Yang[2], Chang-Hao Yang[1,3], Chung-May Yang[1,3], Fung-Rong Hu[1,3], Tzyy-Chang Ho[1,3], Ta-Ching Chen[1,4*]

[1]Department of Ophthalmology, National Taiwan University Hospital, Taipei, Taiwan
[2]Department of Medical Education, National Taiwan University Hospital, Taipei, Taiwan
[3]Department of Ophthalmology, College of Medicine, National Taiwan University, Taipei, Taiwan
[4]Graduate Institute of Clinical Medicine, College of Medicine, National Taiwan University, Taipei, Taiwan

* Corresponding author:
Ta-Ching Chen, MD
12F, No.7, Zhongshan S. Rd., Zhongzheng Dist., Taipei City 10002, Taiwan.
Tel: +886-2-23123456; ext: 63783
Email: tachingchen1@ntu.edu.tw

Supplemental Figure 2. tiff

Fundus autofluorescence and optical coherence tomography images of typical and pericentral types. (A) Typical type (B) Pericentral type. The involvement of the typical type was more extensive than that of the pericentral type, which was peripheral sparing. Moreover, the preserved photoreceptor area is usually presented as a circle or elliptical shape in a typical type, and unstructured in the pericentral type. The length of the intact ellipsoid zone in the superior direction was not equal to that in the inferior direction in retinitis pigmentosa patients with pericentral type.
