# Supplementary material for: The structure–function correlation analysed by OCT and full field ERG in typical and pericentral subtypes of retinitis pigmentosa
Source: Sci Rep. 2021 Aug 19;11:16883. doi: 10.1038/s41598-021-96570-7 (PMC8376926; doi:10.1038/s41598-021-96570-7)
Supplement: Supplementary file 5 — Supplementary Table 1. [file 41598_2021_96570_MOESM5_ESM.pdf]

The structure-function correlation analysed by OCT and full field ERG in typical and pericentral subtypes of retinitis pigmentosa.

Ching-Wen Huang[1], Jung-Je Yang[2], Chang-Hao Yang[1,3], Chung-May Yang[1,3], Fung-Rong Hu[1,3], Tzyy-Chang Ho[1,3], Ta-Ching Chen[1,4*]

[1]Department of Ophthalmology, National Taiwan University Hospital, Taipei, Taiwan
[2]Department of Medical Education, National Taiwan University Hospital, Taipei, Taiwan
[3]Department of Ophthalmology, College of Medicine, National Taiwan University, Taipei, Taiwan
[4]Graduate Institute of Clinical Medicine, College of Medicine, National Taiwan University, Taipei, Taiwan

* Corresponding author:
Ta-Ching Chen, MD
12F, No.7, Zhongshan S. Rd., Zhongzheng Dist., Taipei City 10002, Taiwan.
Tel: +886-2-23123456; ext: 63783
Email: tachingchen1@ntu.edu.tw

Supplemental Table 1. 212 genes tested in our clinical setting

Disease category	Genes
Bardet-Biedl syndrome, AR	*ARL6, BBIP1, BBS1, BBS2, BBS4, BBS5, BBS7, BBS9, BBS10, BBS12, C8orf37, CEP290, IFT172, IFT27, INPP5E, KCNJ13, LZTFL1, MKKS, NPHP1, SDCCAG8, TRIM32, TTC8*
Chorioretinal atrophy or degeneration, AD	*PRDM13, RGR*
Cone or cone-rod dystrophy, AD	*AIPL1, CRX, GUCA1A, GUCY2D, PITPNM3, PROM1, PRPH2, RIMS1, SEMA4A, UNC119*
Cone or cone-rod dystrophy, AR	*ABCA4, ADAM9, ATF6, C21orf2, C8orf37, CACNA2D4, CDHR1, CERKL, CNGA3, CNGB3, CNNM4, GNAT2, KCNV2, PDE6C, PDE6H, POC1B, RAB28, RAX2, RDH5, RPGRIP1, TTLL5*
Cone or cone-rod dystrophy, XL	*CACNA1F, RPGR*
Congenital stationary night blindness, AD	*GNAT1, PDE6B, RHO*
Congenital stationary night blindness, AR	*CABP4, GNAT1, GNB3, GPR179, GRK1, GRM6, LRIT3, RDH5, SAG, SLC24A1, TRPM1*
Congenital stationary night blindness, XL	*CACNA1F, NYX*
Deafness alone or syndromic, AD	*WFS1*
Deafness alone or syndromic, AR	*CDH23, CIB2, DFNB31, MYO7A, PCDH15, USH1C*
Leber congenital amaurosis, AD	*CRX, IMPDH1, OTX2*
Leber congenital amaurosis, AR	*AIPL1, CABP4, CEP290, CRB1, CRX, DTHD1, GDF6, GUCY2D, IQCB1, KCNJ13, LCA5, LRAT, NMNAT1, PRPH2, RD3, R DH12, RPE65, RPGRIP1, SPATA7, TULP1*
Macular degeneration, AD	*BEST1, C1QTNF5, ELOVL4, FSCN2, GUCA1B, HMCN1, IMPG1, OTX2, PRDM13, PROM1, PRPH2, RP1L1, TIMP3*
Macular degeneration, AR	*ABCA4, DRAM2, IMPG1*
Macular degeneration, XL	*RPGR*
Age-related macular degeneration (AMD)	*ABCA4 FBLN5, HMCN1, RAX2*

Retinitis pigmentosa, AD	*BEST1, CA4, CRX, FSCN2, GUCA1B, HK1, IMPDH1, IMPG1, KLHL7, NR2E3, NRL, PRPF3, PRPF4, PRPF6, PRPF8, PRPF31, PRPH2, RDH12, RHO, ROM1, RP1, RP9, RPE65, SAG, SEMA4A, SNRNP200, TOPORS*
Retinitis pigmentosa, AR	*ABCA4, AGBL5, ARL6, ARL2BP, BBS1, BBS2, BEST1, C2orf71, C8orf37, CERKL, CLRN1, CNGA1, CNGB1, CRB1, CYP4V2, DHDDS, DHX38, EMC1, EYS, FAM161A, HGSNAT, IDH3B, IFT172, IMPG2, KIAA1549, KIZ, LRAT, MAK, MERTK, MVK, NEK2, NEUROD1, NR2E3, NRL, PDE6A, PDE6B, PDE6G, PRCD, PROM1, RBP3, RGR, RHO, RLBP1, RP1, RP1L1, RPE65, SAG, SAMD11, SLC7A14, SPATA7, TTC8, TULP1, USH2A, ZNF408, ZNF513*
Retinitis pigmentosa, XL	*OFD1, RP2, RPGR*
Syndromic/systemic diseases with retinopathy, AD	*ABCC6, ATXN7, COL11A1, COL2A1, JAG1, KCNJ13*
Syndromic/systemic diseases with retinopathy, AR	*ABCC6, ABHD12, ACBD5, ADAMTS18, AHI1, ALMS1, CC2D2A, CEP290, COL9A1, CSPP1, ELOVL4, FLVCR1, GNPTG, HARS, HGSNAT, INPP5E, INVS, IQCB1, LAMA1, LRP5, NPHP1, NPHP3, NPHP4, PANK2, PCYT1A, PEX1, PEX2, PEX7, PHYH, PNPLA6, POC1B, PRPS1, RDH11, RPGRIP1L, SDCCAG8, TMEM216, TMEM237, TTPA, TUB, WDPCP, WFS1, ZNF423*
Syndromic/systemic diseases with retinopathy, XL	*OFD1,*
Usher syndrome, AR	*ABHD12, CDH23, CEP250, CIB2, CLRN1, DFNB31, HARS, MYO7A, PCDH15, USH1C, USH1G, USH2A*
Other retinopathy, AD	*BEST1, CRB1, FZD4, ITM2B, LRP5, MAPKAPK3, MIR204, OPN1SW, RCBTB1, TSPAN12, ZNF408*
Other retinopathy, AR	*ASRGL1, BEST1, CDH3, CNGA3, CNGB3, CNNM4, CYP4V2, LRP5, MFRP, MVK, NR2E3, OAT, PROM1, RCBTB1, RLBP1*
Other retinopathy, XL	*CACNA1F, CHM, NDP, OPN1LW, OPN1MW, PGK1*
Other putative genes	*ADGRA3, ARL13B, CLRN3, COL11A2, GPR143, OR2W3, PEX26, TMEM67, RS1*
Macular degeneration, AD	*BEST1, C1QTNF5, ELOVL4, FSCN2, GUCA1B, HMCN1, IMPG1, OTX2, PRDM13, PROM1, PRPH2, RP1L1, TIMP3*

AD, autosomal dominant; AR, autosomal recessive; XL, X-linked
